# The Impact of a Case of Ebola Virus Disease on Emergency Department Visits in Metropolitan Dallas-Fort Worth, TX, July, 2013–July, 2015: An Interrupted Time Series Analysis

**DOI:** 10.1371/currents.outbreaks.e62bdea371ef5454d56f71fe217aead0

**Published:** 2018-03-20

**Authors:** Noelle-Angelique M. Molinari, Tanya Telfair LeBlanc, William Stephens

**Affiliations:** Office of Public Health Preparedness and Response, Centers for Disease Control and Prevention, Atlanta, Georgia, USA; Applied Science and Evaluation Branch, Office of Public Health Preparedness and Response, Centers for Disease Control and Prevention, Atlanta, Georgia, USA; Office of Public Health Informatics, Tarrant County Public Health, Tarrant County, Texas, USA

**Keywords:** Ebola Virus Disease, emergency department visits, statistical model

## Abstract

**Background:**

The first Ebola virus disease (EVD) case in the United States (US) was confirmed September 30, 2014 in a man 45 years old. This event created considerable media attention and there was fear of an EVD outbreak in the US.

**Methods:**

This study examined whether emergency department (ED) visits changed in metropolitan Dallas-Fort Worth­­, Texas (DFW) after this EVD case was confirmed. Using Texas Health Services Region 2/3 syndromic surveillance data and focusing on DFW, interrupted time series analyses were conducted using segmented regression models with autoregressive errors for overall ED visits and rates of several chief complaints, including fever with gastrointestinal distress (FGI). Date of fatal case confirmation was the “event.”

**Results:**

Results indicated the event was highly significant for ED visits overall (P<0.05) and for the rate of FGI visits (P<0.0001). An immediate increase in total ED visits of 1,023 visits per day (95% CI: 797.0, 1,252.8) was observed, equivalent to 11.8% (95% CI: 9.2%, 14.4%) increase ED visits overall. Visits and the rate of FGI visits in DFW increased significantly immediately after confirmation of the EVD case and remained elevated for several months even adjusting for seasonality both within symptom specific chief complaints as well as overall.

**Conclusions:**

These results have implications for ED surge capacity as well as for public health messaging in the wake of a public health emergency.

## Background and Rationale

Ebola virus disease (EVD) is a rare but deadly and highly contagious zoonotic infection first recognized in 1976 in rural Central Africa.^1^^,^^2^ Though associated with high mortality, the remote locations characterized by low population density of these early outbreaks, curtailed extensive exposure.^1^ In stark contrast, the 2014 EVD outbreaks in West Africa (Guinea, Liberia, and Sierra Leone) occurred primarily in urban areas. 1^,^^2^ ^3^ Widespread morbidity and mortality are associated with the hemorrhagic fever caused by the virus. An unprecedented number of cases (>28,600) were reported since the first confirmed case in March 2014.^3^^,^^4^ Among clinical cases, 2 in 5 died from EVD.^4^^,^^5^ With thousands of persons at-risk for EVD and an overwhelmed health care system, the World Health Organization declared the West African outbreak an epidemic in March 2014^5^ ^5,6^ and in August 2014 a Public Health Emergency of International Concern.^1^

In an international effort to assist affected countries, and reduce the likelihood of disease spread, a number of countries supplied humanitarian aid including volunteers, medical aid, and sanitation equipment to affected countries.^3^^,^^5^^,^^6^ Clinicians, epidemiologists, and other health care providers, as well as business and personal travelers to and from the epidemic epicenters heightened the concern for global transmission of EVD.^5^ ^6^ In response, the World Health Organization, the United States Department of Health and Human Services (HHS), the Centers for Disease Control and Prevention (CDC), and other international partners implemented measures to rapidly screen, detect, and treat travelers entering non-epidemic countries.^7^^,^^8^ Consequently, risk of EVD exposure in persons returning home after serving in outbreak endemic areas fueled fear of an epidemic in the United States.^7^ ^8^

Despite best efforts to screen persons entering the United States from these West African countries, a person entered the United States from Liberia on September 20, 2014 with undetected EVD. The patient presented at a Dallas, Texas hospital emergency department (ED) on September 25, 2014 and was treated for sinusitis and released. Several days later, the patient returned to the hospital and EVD diagnosis was confirmed on September 30, 2014, hereafter referred to as the event.^6^ ^9^ The patient died on October 8, 2014 (herein referred to as the primary case). Two nurses who provided direct care for the primary case also were confirmed with EVD on October 11, 2014 and October 15, 2014.^6^^,^^7^ ^9^ These events created considerable media attention, and there was fear of an EVD outbreak in the United States. In the state of Texas, where the case fatality occurred, the perception of a widespread outbreak threat was plausibly magnified.

In this paper, we examine whether the report of a confirmed fatal case of EVD in the Dallas-Fort Worth (DFW), Texas metropolitan area impacted the number and type of emergency department (ED) visits in that same area. We used a particular statistical methodology, Interrupted Time Series (ITS), to determine if this highly publicized EVD case affected the number of ED visits, overall and for several chief complaints at presentation. The results can be used by public health professionals to assess the potential impact on the demand for emergency medical care in the event of highly publicized cases of infectious diseases, as well as the suitability of this statistical methodology (ITS) to evaluate the impact of similar events in the future.

## Methods

**Data Sources & Measurement**

Data for this study were compiled from the Texas Health Services Region 2/3 syndromic surveillance data and associated ESSENCE analytics through the North Texas Syndromic Surveillance System during the period July 21, 2013–22, 2015. The data captured for the DFW metro area included all contributing EDs from Collin, Dallas, Denton, and Tarrant counties. Note that secondary data, collected through the aforementioned syndromic surveillance system, was analyzed for this study, focusing on data from the region of north Texas where the EVD case was located. The data were aggregated into daily counts of visits by chief complaint in ESSENCE and then exported for analysis in SAS v9.3. These data are classified as a "Limited Data Set" under the Health Insurance Portability and Accountability Act of 1996 (HIPAA) which is to be protected as personal health information. The data use agreements only allow data sharing if it is aggregated and fully de-identified as presented in this paper. As surveillance data, the system does not constitute human subjects research and is exempted from IRB. No interviews were conducted and no informed consent was obtained as human subjects protection procedures were not required. Our research design was reviewed by the CDC Office of Public Health Emergency Preparedness and Response Human Subjects Protection Coordinator and found to be exempt, Human Subjects Research #17121901.

ITS models were specified as segmented regressions with autoregressive errors. ED visits were modeled, overall and for several chief complaints, including fever and fever with gastrointestinal distress. On September 30, 2014, EVD was confirmed in a patient in Dallas, Texas. The date of this fatal case defines the event hypothesized to interrupt the daily time series of ED visits. Visits and visit rates by chief complaint before and after the event were analyzed and absolute and relative effects of the event were estimated.

Daily counts for all ED visits (ALL) and daily rates of ED visits for each of 5 chief complaint categories were examined including: 1) chief complaints of fever (FEV); 2) chief complaints of fever with no mention of cold or flu (FNF); 3) chief complaints of fever plus gastrointestinal symptoms (FGI); 4) chief complaints of fever plus travel to West Africa (FTWA); and 5) chief complaints of asthma (Asthma) which served as a quasi-control group that should not be mistaken for symptoms consistent with EVD. Chief complaint categories 1 through 4 represent increasing specificity toward the EVD symptom profile; the index case was included in FGI cases. Rates of visits were defined as the number of visits for that chief complaint per 1,000 ED visits for all complaints. Thus, the rate of fever visits was defined as the number of visits for chief complaint of fever divided by the number of ED visits for all complaints in the DFW Metro area and multiplied by 1,000.

**Statistical Methods**

Descriptive statistics of mean, standard deviation, and range were calculated for the outcomes in each chief complaint category before and after the event date. Each of the time series was examined visually and trend and seasonal statistics were analyzed by outcome. There were 732 days in each time series with 436 days pre-event (July 21, 2013–September 29, 2014) and 296 days post-event (September 30, 2014–July 22, 2015). All statistical analyses were conducted using the statistical software package SAS v9.3.

ITS models of daily ED visit counts and rates in DFW for each of the outcomes previously enumerated were estimated via maximum likelihood. These models were specified as linear segmented regressions with autoregressive errors.^9^ ^10^^,^^11^ The resulting estimated parameters and standard errors were used to produce estimates and associated 95% confidence intervals of the event-attributable number and percentage change in visits at several time points post-event, as well as the total number of excess visits attributable to the event and the date at which visits and visit rates returned to normal, or were not significantly different from their predicted level in the absence of the event. Detailed description of methods is presented in the Technical Appendix.

## Results

During the observation period before the event (July 21, 2013–September 29, 2014), among 44 hospitals that contributed data in the DFW metropolitan area, the mean number of ED visits per day was 8,875 (SD=1380.5). After September 30, 2014 when the EVD case was confirmed, ED visits increased (µ = 9,439.9, SD=1328.4) and remained higher on average compared to pre-event levels, even during corresponding seasonal surge periods related to expected cold and flu activity. After the event (post-event period, July 21, 2013–July 22, 2015), the mean number of ED visits remained higher than pre-event levels until June, 2015 (see Table 1).

**Figure:**
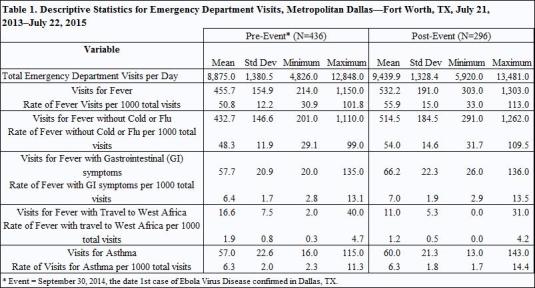

Total ED visits (ALL) in DFW averaged ~ 8,875 per day (range = 4,826–12,848) prior to the event, with peak occurring during January 2014. Post-event, total ED visits averaged ~ 9,440 (range= 5,920–13,481), with peak occurring during December 2014. Visits for FEV accounted for about 456 visits per day (range=214–1,150) prior to the event with peak occurring from December 2013–January 2014. FEV visits averaged ~532 (range=303–1,303) post-event, the peak occurring in December 2014. Daily visits for Asthma were similar to FGI visits prior to the event (~57 visits per day) but diverged somewhat post-event with FGI visits increasing to 66 visits per day on average while Asthma visits increased to 60 visits per day on average. Peaks for FGI visits occurred in December each year while peaks for Asthma visits occurred in February and September 2014 pre-event and February through March 2015 post-event. Visits for FTWA were least common, averaging about 16 per day pre-event and declining to 11 visits per day post-event. Peak visits for FTWA occurred prior to April 2014 and FTWA visits declined throughout the remainder of the study period.

Results from the ITS regression models indicated that the event was significant for ALL visits (P<0.05) and for the rate of FGI visits (P<0.0001), representing a significant interruption to these time series. For both chief complaint categories, average visits and visit rates increased immediately following the event and then gradually declined, eventually approaching the pre-event baseline. The event effect was significant at P<0.10 for the rate of FTWA and for the rate of FEV visits. The event was not significant for the rates of FNF visits and Asthma visits. The rate of FTWA visits displayed a steady downward trend throughout the study period. See Technical Appendix.

**Figure:**
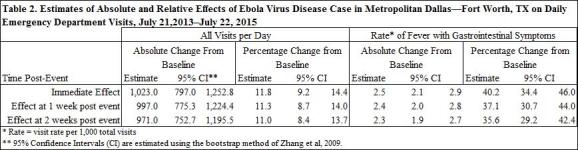

Table 2 displays the estimated absolute and relative effects on ED visits of the EVD case diagnosed on September 30, 2014 in Dallas, TX. The ITS analyses indicated that this event resulted in an immediate increase in ALL visits of 1,023.0 visits per day (95% CI: 797.0, 1,253.8). This is equivalent to an immediate relative increase of 11.8% (95% CI: 9.2%, 14.4%) in ALL visits. Although FGI visit rates increased by 40.2% (95% CI: 34.4%, 46.0%), only a small portion of the increase in ALL visits, 2.5 (95% CI: 2.1, 2.9) visits per 1,000 or about 20 visits per day, was due to the increase in FGI visit rates. This increase was sustained; at 14 days post-event, ALL visits were still elevated by 971.0 (95% CI: 752.7, 1195.5). The rate of FGI visits also remained elevated by 35.5% (95% CI: 29. 2%, 42. 4%) at 14 days post-event.

Beginning on the date that the EVD case was diagnosed and until the change in ALL visits was insignificant, January 22, 2015, the total impact of the EVD case on ALL visits was 95,690 (95% CI: 69,185, 116,202) excess visits. The total impact of the EVD case on FGI visits, which returned to baseline by March 20, 2015, was 2,151 (95% CI: 2055, 2247) excess FGI visits.

## Discussion

This study found that ED visits in the Dallas-Fort Worth, Texas metropolitan area increased significantly in both absolute and relative terms immediately after confirmation of the fatal EVD case. Visits for ALL complaints increased by about 12% immediately (~1,123 visits per day) and remained elevated by over 11% (~971 visits per day) at two weeks post-event, returning to baseline on January 22, 2015. The rate of visits for fever with gastrointestinal distress increased by over 40% (~2.5 per 1000 visits per day) immediately and remained elevated by over 35% (~2.3 per 1000 total visits per day) at two weeks post event and returned to baseline on March 20, 2015. Visits for fever with travel to West Africa declined throughout the study period. It is worth noting that while there was an observed increase in ED visits with chief complaints that aligned with EVD-like illness, none of the observed increase in FGI was linked to a confirmed case of EVD and the majority of the observed increase in ED visits occurred outside the EVD-specific symptom profile.

Though this study does not prove unequivocally that the fatal EVD case caused a surge in ED visits, a clear pattern of association is observed and it does establish preliminary evidence of causality. Interrupted Time Series analysis is a robust modelling technique; the strongest quasi-experimental method for evaluating the longitudinal effects of an event.^10^ Our use of segmented regression with autoregressive errors provides a powerful method for estimating the impact of the event while adjusting for secular events.^11^ To our knowledge, this is the first application of ITS analysis to a public health emergency event.

The main threat to validity using this method is the potential for confounding effects due to a co-occurring or secular event. Despite concerted search and consult with local experts, the authors did not uncover any event that occurred in the DFW metro area at or near the time of the EVD case that could have affected the number and type of ED visits. The statistical methods employed address secular changes through the use of a comparison group, specifically ED visits for asthma. Asthma visits should not have been mistaken for EVD and did not display a significant increase during the period following the event. Statistical methods also addressed autocorrelation and seasonality, such as seasonal influenza cases, via estimation of autoregressive errors. There was no indication that the population of DFW changed significantly during the study period, nor did the coding of chief complaints for ED visits.

These results have implications for ED surge capacity as well as for public health messaging in the wake of a public health emergency. Previous disaster research suggests that EDs do experience surge following disasters, both natural and man-made.^12^^,^^13^^,^^14^^,^^15^ Results presented here are consistent with these findings. However, this study indicates most of the surge in ED visits did not involve Ebola-specific symptoms and none of the observed increase in cases following the event actually involved EVD since the outbreak was contained. Previous research indicated that mass media, especially internet and social networking, play an important role in interpreting and disseminating health news to the public.^16^^,^^17^ This suggests a need for targeted public health messaging to improve public understanding of disease symptoms as well as allay concerns. Public health professionals should consider the implications of messaging and communications regarding rare, deadly, and highly contagious diseases of high profile such as EVD.

## Technical Appendix

**Statistical Methods: Interrupted Time Series**

ITS models of daily ED visit counts and rates in DFW for each of the outcomes previously enumerated were estimated via maximum likelihood. ITS models were specified as linear segmented regression with autoregressive errors. Time series were tested for stationarity via the augmented Dickey-Fuller test and autocorrelation via generalized Durbin-Watson tests. Autoregressive error models were used to address autocorrelation and seasonality where present. Models were estimated in SAS using “proc autoreg” with autoregressive error terms identified via backward elimination of lags where p>0.05.^9^^,^^10^^,^^11^

The ITS is modeled using the following linear segmented regression with autoregressive errors, which represents the baseline level and trend of the outcome variables before the event and changes in the level and trend after the event for each outcome:

**Figure:**
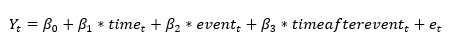
(1)

Here, \begin{equation*}Y_{t}\end{equation*} represents the dependent variable at a point in time, either daily ED visits or daily ED visit rates by chief complaint. \begin{equation*}time_{t}\end{equation*} is the continuous variable representing time in days since the beginning of the study period. The intervention function was specified as a step function. \begin{equation*}event_{t}\end{equation*} is an indicator variable set to zero prior to the date of the event and becoming 1 on the day of the event and for the duration of the time series after the event. \begin{equation*}timeafterevent_{t}\end{equation*} is a continuous variable counting the days elapsed since the event at time t and set to zero prior to the event. The regression error, \begin{equation*}e_{t}\end{equation*} , is comprised of a random error component as well as an autoregressive error component to adjust for autocorrelation and seasonality.

Then the following represents the estimated factual case:

**Figure:**
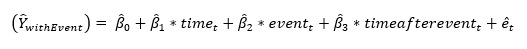
(2)

The estimated counterfactual case is represented as:

**Figure:**
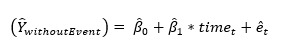
(3)

Note that the parameter estimate, \begin{equation*}\beta _{0}\end{equation*}, is the baseline level of the outcome variable at time zero, or the intercept. \begin{equation*}\beta _{1}\end{equation*} is the estimated baseline trend of the outcome variable, or the daily deviation from the baseline level prior to the event. \begin{equation*}\beta _{2}\end{equation*} is the estimated absolute change in level, or intercept, of the outcome that occurs immediately following the event and \begin{equation*}\beta _{3}\end{equation*} is the estimated absolute change in trend that occurs after the event. The estimated relative, or percentage, change in the baseline level due to the event is \begin{equation*}(\beta _0+\beta _2)/\beta _0 \end{equation*}, while the estimated relative change in trend is \begin{equation*}(\beta _1+\beta _3)/\beta _1 \end{equation*}. Thus, \begin{equation*}\beta _0+\beta _2\end{equation*} is the estimated post-event baseline of the outcome variable and \begin{equation*}\beta _1+\beta _3\end{equation*} is the estimated post-event trend in the outcome variable. The estimated absolute impact of the event measured one week post-event is equation (2) minus equation (3) evaluated at t=452, or \begin{equation*}(\beta _1+\beta _3*7)\end{equation*}. And the estimated relative impact of the event measured one week post-event is \begin{equation*}(\beta _2+\beta _3*7)/(\beta _0+\beta _1*452)\end{equation*}.

Estimates of the parameters and standard errors retrieved from equation (2) were used to produce estimates and associated 95% confidence intervals of the estimated absolute and relative changes in level and trend as well as the estimated absolute and relative impact of the event at one and two weeks post-event in terms of the outcome variable. The total impact of the event was calculated as the sum of the estimated absolute impact of the event over time, until the absolute impact became insignificant. Based on the findings of Zhang, et al. 2009, the 95% confidence intervals of the estimated absolute and relative changes for baseline, trend, and the various time points post-event were obtained by bootstrapping. Specifically, parameter estimates from equation (2) for each of the study outcomes were retrieved and used to simulate 10,000 observations based on these estimates assuming normally distributed errors. The bootstrapped 95% confidence intervals were constructed from the 2.5 and 97.5 percentiles of this simulated data.^11^

Equation (2) was estimated for each of the six outcomes (ALL, FEV, FNF, FGI, FTWA, and Asthma) and graphs were produced, displaying \begin{equation*}(Y_{withEvent} )\end{equation*}, \begin{equation*}(Y_{withEvent})-e_t\end{equation*}, and the observed data over the study period by outcome. The parameter estimates from equation (2) for each outcome with significant \begin{equation*}\beta _2\end{equation*} were used to produce equation (3). The interruption observed in \begin{equation*}(Y_{wuthEvent}) - e_{t}\end{equation*}, the predicted trend line, represents the event effect. Finally, \begin{equation*}(Y_{withEvent})\end{equation*}, \begin{equation*}(Y_{withEvent})-e_t\end{equation*}, \begin{equation*}(Y_{withoutEvent})\end{equation*}, and \begin{equation*}(Y_{withoutEvent})-e_t\end{equation*} were displayed graphically over the study period. The sum of the differences between \begin{equation*}(Y_{withEvent})-e_t\end{equation*} and \begin{equation*}(Y_{withoutEvent})-e_t\end{equation*} was the total impact of the event.

**Results**

During the observation period before the event (July 21, 2013–September 29, 2014), among 44 hospitals that contributed data in the DFW metropolitan area, the mean number of ED visits per day was 8,875 (SD=1380.5). After September 30, 2014 when the EVD case was confirmed, ED visits increased (µ = 9,439.9, SD=1328.4) and remained higher on average compared to pre-event levels, even during corresponding seasonal surge periods related to expected cold and flu activity. After the event (post-event period, July 21, 2013–July 22, 2015), the mean number of ED visits remained higher than pre-event levels until June, 2015.

Total ED visits (ALL) in DFW averaged ~ 8,875 per day (range = 4,826–12,848) prior to the event, with peak occurring during January 2014. Post-event, total ED visits averaged ~ 9,440 (range= 5,920–13,481), with peak occurring during December 2014. Visits for FEV accounted for about 456 visits per day (range=214–1,150) prior to the event with peak occurring from December 2013–January 2014. FEV visits averaged ~532 (range=303–1,303) post-event, the peak occurring in December 2014. Daily visits for Asthma were similar to FGI visits prior to the event (~57 visits per day) but diverged somewhat post-event with FGI visits increasing to 66 visits per day on average while Asthma visits increased to 60 visits per day on average. Peaks for FGI visits occurred in December each year while peaks for Asthma visits occurred in February and September 2014 pre-event and February through March 2015 post-event. Visits for FTWA were least common, averaging about 16 per day pre-event and declining to 11 visits per day post-event. Peak visits for FTWA occurred prior to April 2014 and FTWA visits declined throughout the remainder of the study period.

**Figure:**
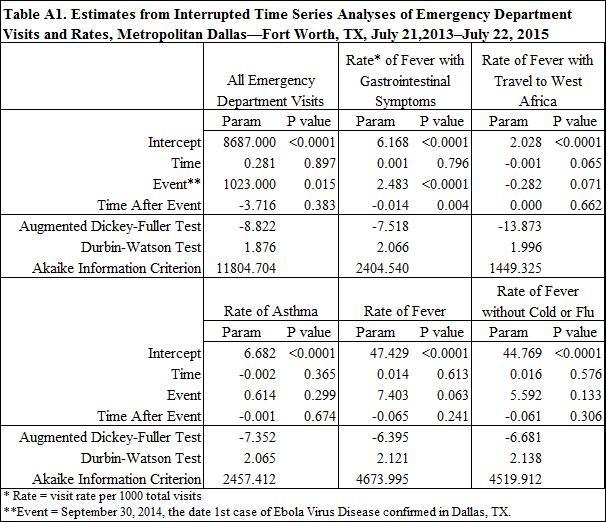

Results from the ITS segmented regression models with autoregressive errors are presented in Table A1 and Figure A1 The event was highly significant for ALL visits (P<0.05) and for the rate of FGI visits (P<0.0001). The coefficient estimate on TimeAfterEvent, which represents the change in the slope after the event, was also significant for the rate of FGI visits (P<0.05). Thus, average visits and visit rates increased immediately following the event and then gradually declined, eventually approaching the pre-event baseline. The event effect was significant at P<0.10 for the rate of FTWA and for the rate of FEV visits. The event was not significant for the rates of FNF visits and Asthma visits.

**Figure:**
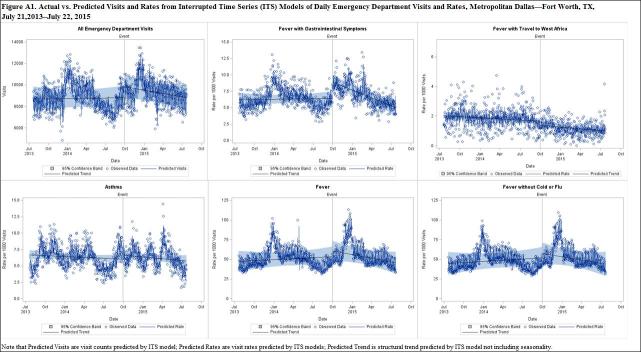

In Figure A1, the interruption in the trend line represents the effect of the event. A significant interruption is observed for ALL visits as well as the rate of FGI visits, followed by a change in the slope of the trend line. Less significant interruptions are observed in the rates of FEV and FTWA. It is worth noting that the rate of FTWA visits displays a fairly steady downward slope during the study period. This is also observed in the negative coefficient estimate on time displayed in Table 2, although it is not highly significant (P=0.07).

**Figure:**
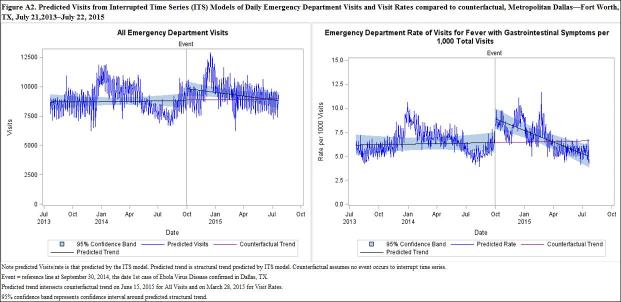

Figure A2 depicts ALL visits and FGI visit rates predicted by the ITS models in which the event occurs compared to the counterfactual . Note that the black trend line in the case of the event veers away from the counterfactual trend line (purple) but eventually returns to close the gap before the end of the study period. ALL visits was not significantly different from baseline by January 22, 2015, with a total impact of 95,690 (95% CI: 69,185, 116,202) excess visits.ALL visits did not drop below the pre-event baseline until June, 2015. FGI visit rates returned to baseline on March 20, 2015 with a total impact of 2,151 (95% CI: 2055, 2247) excess visits.

## Funding

The authors received no specific funding for this work.

## Competing Interests

The authors have declared that no competing interests exist.

## Data Availability

Data is owned by 106 hospital facilities in Health Service Region 2/3 of north Texas who provide syndromic surveillance data strictly for purposes of public health surveillance under provisions of the HITECH Act 2009 (meaningful use), specifically for research and preparedness purposes. Formal Data Use Agreements (DUAs) are in place with every hospital that do not allow public disclosure of detailed datasets used in this study which identify visit records with a particular hospital or patient location. The DUA’s are formal contracts between hospital and Tarrant County as the governmental agency collecting data and operating a state syndromic surveillance system. The DUA’s are in place to protect both patient and hospital sensitive data from public disclosure as the data is classified as a “Limited Data Set” under the Health Insurance Portability and Accountability Act of 1996 (HIPAA) which is to be protected as personal health information (PHI). DUA’s only allow data sharing if it is aggregated and fully de-identified as presented in this paper. Posting detailed data online or providing access to the detailed data that allows replication of this study, or other use of the data to non-public health personnel constitutes a legal violation of these DUA’s.

For requests regarding this data, please contact:

William F. Stephens, MS

Informatics Manager

Office of Public Health Informatics

1101 S. Main

Fort Worth, TX 76104

(817) 321-5365

wfstephens@tarrantcounty.com

## Corresponding Author

Noelle-Angelique M. Molinari, PhD

nmolinari@cdc.gov
